# Novel antigens in non-small cell lung cancer: SP17, AKAP4, and PTTG1 are potential immunotherapeutic targets

**DOI:** 10.18632/oncotarget.2802

**Published:** 2014-11-16

**Authors:** Leonardo Mirandola, Jose A. Figueroa, Tam T. Phan, Fabio Grizzi, Minji Kim, Rakhshanda Layeequr Rahman, Marjorie R. Jenkins, Everardo Cobos, Cynthia Jumper, Raed Alalawi, Maurizio Chiriva-Internati

**Affiliations:** ^1^ Division of Hematology & Oncology and Southwest Cancer Treatment and Research Center, Texas Tech University, Lubbock, TX, USA; ^2^ Laura W. Bush Institute for Women's Health and Center for Women's Health and Gender-Based Medicine, Amarillo, TX, USA; ^3^ Kiromic, LLC. Lubbock, TX, USA; ^4^ Humanitas Clinical and Research Center, Milano, Italy; ^5^ Division of Surgical Oncology, Texas Tech University Medical Center, Amarillo, TX, USA; ^6^ Division of Pulmonary and Critical Care Medicine, Department of Internal Medicine, Texas Tech University Health Sciences Center, Lubbock, TX, USA

**Keywords:** cancer/testis antigens, lung cancer, cancer vaccines

## Abstract

Lung cancer is the leading cause of cancer deaths in both genders worldwide, with an incidence only second to prostate cancer in men and breast cancer in women. The lethality of the disease highlights the urgent need for innovative therapeutic options. Immunotherapy can afford efficient and specific targeting of tumor cells, improving efficacy and reducing the side effects of current therapies. We have previously reported the aberrant expression of cancer/testis antigens (CTAs) in tumors of unrelated histological origin. In this study we investigated the expression and immunogenicity of the CTAs, Sperm Protein 17 (SP17), A-kinase anchor protein 4 (AKAP4) and Pituitary Tumor Transforming Gene 1 (PTTG1) in human non-small cell lung cancer (NSCLC) cell lines and primary tumors. We found that SP17, AKAP4 and PTTG1 are aberrantly expressed in cancer samples, compared to normal lung cell lines and tissues. We established the immunogenicity of these CTAs by measuring CTA-specific autoantibodies in patients' sera and generating CTA-specific autologous cytotoxic lymphocytes from patients' peripheral blood mononuclear cells. Our results provide proof of principle that the CTAs SP17/AKAP4/PTTG1 are expressed in both human NSCLC cell lines and primary tumors and can elicit an immunogenic response in lung cancer patients.

## INTRODUCTION

Lung cancer is the leading cause of cancer-related deaths worldwide, with approximately 226,160 new cases and 160,340 deaths in the Unites States in 2013[1]. Non-small cell lung carcinoma (NSCLC) accounts for approximately 85% of diagnosed lung cancers and is associated with an overall 5-year survival rate of less than 20%[1]. Despite advances in the diagnosis and treatment of patients with NSCLC, the vast majority of patients succumb to the disease. Therefore, there is an urgent need to develop more effective therapies for patients suffering from this devastating neoplasm.

Immunotherapy has recently emerged as a promising therapy for NSCLC[2]. The generation of immune responses against specific tumor-associated antigens and/or manipulation of T-cell checkpoints to enhance anti-tumor responses are an active area of investigation[2]. Cancer/testis antigens (CTAs) are a family of proteins with testis-restricted expression and negligible expression in normal tissues. We and others have shown that CTAs are frequently expressed in many tumors at the mRNA and protein levels [3-6]. More recently, the CTAs NY-ESO-1 and MAGE-A2/3/4/6 have been detected in NSCLC primary tumors and their immunogenicity suggests they are promising targets for the development of potentially effective lung cancer vaccines [7-9]. We have previously demonstrated that the CTAs SP17, AKAP4, Ropporin, and PTTG1 are potential immunotherapeutic targets in ovarian cancer, multiple myeloma, and prostate cancer [3, 10-12]. In this study we evaluated RNA and protein expression patterns of SP17, AKAP4 and PTTG1 in NSCLC cell lines and primary tumor samples from NSCLC patients, compared to normal lung cells and tissues. We also determined the immunogenicity of these CTAs by measuring CTA-specific antibodies (Abs) in the sera of lung cancer patients and generating CTA-specific cytotoxic anti-tumor responses *in vitro*, using autologous peripheral blood mononuclear cells (PBMCs).

## RESULTS

### SP17/AKAP4/PTTG1 mRNAs are up-regulated in NSCLC primary tumors compared to normal lung tissues

**Figure 1:**
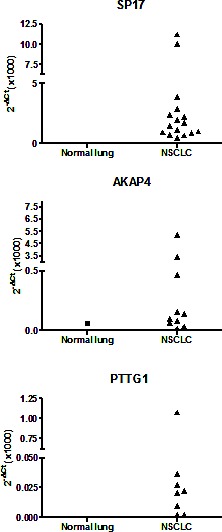
qRT-PCR analysis of CTA expression in cohort 1 Data are expressed as means calculated out of assays run in triplicate. β-actin was used as internal reference to compute the δCt values.

SP17, AKAP4 and PTTG1 expression was determined by qRT-PCR in a panel of cDNAs from NSCLC and normal lung tissues. Figure 1 shows that SP17, AKAP4, and PTTG1 were undetectable in normal lung samples, with the exception of AKAP4 in 1 healthy sample which showed weak expression. Overall, more than 65% (n=26) of NSCLC showed the expression of at least one CTA, while more than 20% (n=8) expressed 2 or more. Forty percent of samples were SP17^+^, 25% were AKAP4^+^, while 20% were PTTG1^+^. There was no association with tumor histological type, grade, or stage.

### SP17/AKAP4/PTTG1 protein expression is present in NSCLC cell lines and primary tumor specimens, but not in normal bronchus epithelium

**Figure 2:**
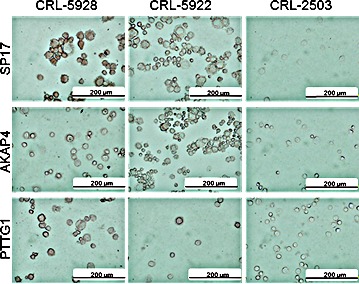
Immunocytochemistry NSCLC cell lines and the non-tumor bronchus epithelium cell line, CRL-2503, were analyzed for CTA expression using DAB staining (brown signal). Pictures were taken using an inverted Olympus X71 microscope.

We explored CTA expression in 2 NSCLC cell lines and 1 immortalized normal bronchus cell line. ICC analysis (Figure 2) shows undetectable expression of the CTA panel in the normal bronchus cell line (CRL-2503), whereas the 3 CTAs were detected in NSCLC cell lines (CRL-5928 and CRL-5922).

**Figure 3:**
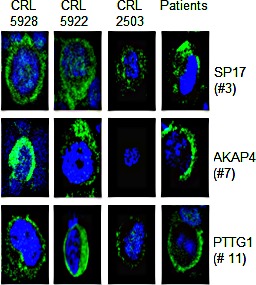
Immunofluorescence Representative IF performed on NSCLC cell lines, CRL-5928, CRL-5922, on the normal bronchus epithelium-derived cells CRL-2503, and representative patients. We show the positive stain for SP17/AKAP4/PTTG1 (green signal) in the cytoplasm. Blue signal (nucleus) = DAPI. Pictures were taken at 60X magnification by an inverted florescence microscope (Olympus IX71).

**Figure 4:**
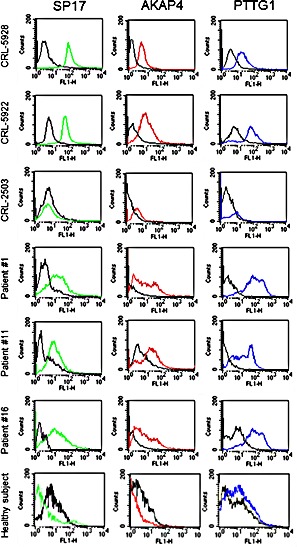
Flow-cytometry analysis Cells were incubated with the indicated antibody (colored histograms) or corresponding isotypic controls (black histograms). Fluorescence intensity was measured on FSC/SSC-gated cells (10,000 events) using a FACS-Canto flow cytometer (BD).

We then validated the specificity of CTA protein expression in normal and primary NSCLC tissues using IF and flow-cytometry. IF (Figure 3) shows that SP17, AKAP4 and PTTG1 are expressed by NSCLC cell lines, but not by the CRL-2503 non-tumorigenic bronchial cell line. Figure 3 also shows representative results from 3 patients (results for all of the patients evaluated in this study are summarized in Table 2). Flow-cytometry (Figure 4) confirmed these results, showing that SP17, AKAP4 and PTTG1 are expressed by NSCLC cell lines, but not by the non-transformed bronchial cell line. Figure 4 also shows representative results from 3 patients. The specificity of CTA expression in NSCLC was confirmed by the absence of positive staining for all CTAs in tissue derived from a healthy individual (a representative result is displayed in Figure 4). Table 2 summarizes the results obtained on the NSCLC cohort. Overall, 52% of samples were SP17^+^, 29% were AKAP4^+^, while 35% were PTTG1^+^.

### Circulating CTA-specific autoantibodies are detectable in the sera of NSCLC patients

**Figure 5:**
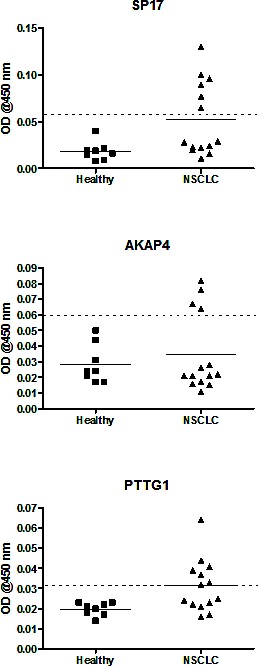
ELISA for the detection of circulating CTA-specific IgG Graphs display mean OD values calculated from experiments run in triplicate. The horizontal dotted line represents the positivity cut-off, calculated as the median value obtained from the healthy control group + 2 standard deviations.

We compared the levels of circulating CTA-specific IgG autoantibodies in sera from NSCLC patients with those from healthy subjects, by indirect ELISA. A subject was considered positive if the ELISA signal was at least 2 Standard Deviations (SD) higher than the median signal found in healthy subjects [13]. Figure 5 shows that the majority of NSCLC patients displayed CTA-specific autoantibodies. Detailed results for each patient are summarized in Table 2.

### Generation of CTA-specific CTLs from patients' PBMCs

**Figure 6:**
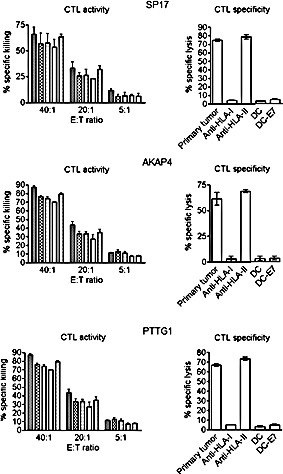
Analysis of CTL activity (left) and specificity (right) The histograms show the percentage of specific lysis obtained through a non-radioactive EUROPIUM-based assay under the indicated different conditions. The left panel shows the CTL activity at different ratios of effector (E, autologous CTL): target (T, autologous primary tumor cells) cells. Bars represent the mean of experiments run in triplicate, and error bars represent standard deviations. The right panel shows the analysis of CTL specificity. Bars represent the mean values obtained from experiments run on the selected 5 patients, while error bars represent standard deviations. Antibodies against HLA class I (W6/32) and HLA class II (L243) were added (to block the MHC-I/CD8 and MHC-II/CD4 interaction respectively), at a concentration of 25 μg/mL. Target specificity was confirmed by a lack of significant response against CTA-negative cells (DC not loaded with CTA or DC presenting the CTA-unrelated antigen, HPV-E7). CTL indicates cytotoxic T lymphocyte; DC, dendritic cell; HLA, human leukocyte antigen; E7, HPV-E7 antigen; PBMCs, peripheral blood mononucleated cell.

**Figure 7:**
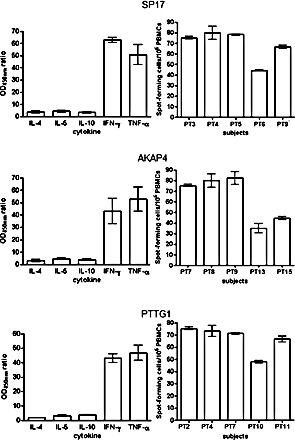
ELISA and ELISPOT Left panel: supernatants from co-cultures (20:1 effector: target ratio) of autologous tumor cells and PBMCs stimulated with CTA-pulsed or unmodified DCs were analyzed for the levels of the indicated cytokines. All measurements were run in triplicate. Results are displayed as the mean ODs measured with activated PBMCs divided by the mean ODs obtained with PBMCs incubated with autologous DCs without antigens (OD_450_ ratio). Right panel: IFN-γ expression by patients' CTLs co-cultured with autologous tumor cells (20:1 effector:target ratio) was evaluated using ELISPOT assay as detailed in the Materials and Methods section. Spot counts were performed with an AID ELISPOT Reader System (Cell Technology, Inc., Columbia, MD) and normalized per 10^6^ PBMCs. Results represent the means ± standard deviations of assays run in triplicate.

We generated CTLs from 5 patients displaying SP17, AKAP4 and PTTG1 protein expression and demonstrated specific lysis of autologous tumor cells and CTA-expressing cell lines (Figure 6). CTL lysis of NSCLC cells derived from patient samples was observed at different effector/target cell ratios (Figure 6, left panel). CTA specificity of CTL-dependent NSCLC cell lysis was confirmed when NSCLC cells were exposed to CTLs generated using non-pulsed DCs and DCs pulsed with HPV-E7 antigen (Figure 6, right panel). HLA class I restriction was evidenced by the inhibition of cytotoxic effect with HLA-I blocking antibody, but not HLA-II blockade (Figure 6, right). Th1 polarization was demonstrated after stimulation with CTA-pulsed DCs and co-culture with tumor cells, by detecting more than 50-fold increase in IFN-γ and TNF-α production and no significant change in IL-4, IL-5, and IL-10 levels, using ELISA (Figure 7, left panel). The ELISPOT for IFN-γ further confirmed the increased in IFN-γ-producing cells derived from PBMCs activated with CTA-presenting DCs, and co-cultured with tumor cells (Figure 7, right panel).

## DISCUSSION

Although NSCLC has historically been considered a weakly immunogenic malignancy[8], several studies have shown it is possible to generate an effective immune response using a DC-based approach [14, 15]. Furthermore, tumor-specific T-cells have been detected in patients with NSCLC [15], indicating identification of specific NSCLC-associated antigens, and presentation to the immune system in an optimal fashion may generate tumor-specific CTLs against this disease [16, 17]. The potential role of immunotherapy in NSCLC patients has been demonstrated by a recent meta-analysis of 12 clinical vaccine trials [17]. When developing cancer immunotherapy, ideal targets for this approach should elicit strong immunogenicity and demonstrate selective expression in cancer cells, but not in normal tissues [2]. CTAs are particularly attractive in this regard based on their tumor-restricted expression pattern and immunogenicity [4, 6], and indeed there is a considerable effort ongoing to develop high throughput techniques, such as bioinformatic meta-analyses [18], for the identification of novel CTAs that are likely clinically relevant as cancer biomarkers and immunotherapy targets.

Here we provide “proof-of principle” for the use of three CTAs, namely SP17, AKAP4, and PTTG1, in the development of NSCLC immunotherapy. We analyzed the differential expression of these CTAs in NSCLC primary tumors, NSCLC cell lines, in healthy lung epithelium samples and in one non-tumorigenic, normal bronchus cell line. NSCLC cell lines expressed SP17/AKAP4/PTTG1 transcripts and proteins, while the non-tumorigenic cell line showed only weak SP17 and PTTG1 expression in immunofluorescence but was negative by immunocytochemistry, possibly due to the higher sensitivity of immunofluorescence. Although we are aware that the data on protein expression is limited to a normal bronchus cell line, this finding is consistent with our previous observations showing that SP17 is expressed in ciliated somatic epithelia, including those of the airways [19]. Moreover, the CRL-2503 is a bronchial epithelial cell line “immortalized” by transfection with the SV40 T antigen, a genetic alteration with unknown effects on SP17 expression. The expression of SP17 RNA by a non-neoplastic bronchial cell line raises the question of potential broncho-pulmonary toxicity when targeting this specific CTA. Nevertheless, preclinical studies using SP17-directed immunotherapies have not demonstrated any toxicity to ciliated cells [11, 20, 21]. Therefore, these observations indicate the level of SP17 expression in these cells is not sufficient to elicit a cytotoxic response. We analyzed two cohorts of NSCLC and normal pulmonary bronchus: one (n=48, 40 NSCLC samples, 8 normal lung) was commercially available (OriGene) and was analyzed by qRT-PCR, while the second cohort (n=25, 17 NSCLC samples and 8 normal lung) was analyzed by flow-cytometry, immunofluorescence, and ELISA. Overall, qRT-PCR data and flow-cytometry/immunofluorescence analysis showed comparable CTA expression frequencies in tumor samples, while the CTAs were undetectable in normal lung samples. In the second cohort, CTA protein expression was confirmed independently by flow-cytometry and immunofluorescence, with consistent results. Of note, more than 88% of tumors showed expression of at least one out of three CTAs studied, while 29% expressed two CTAs, suggesting their relevance as potential therapeutic targets in this disease. Such broad expression could potentially be further expanded with pharmacological treatment based on de-methylating agents: Li B et al. [22] recently showed that CTAs expression can be induced in tumors showing low CTA positivity by 5-aza-2′-deoxycytidine. Of note, they correlated such CTA induction with specific CD8+ T-cell response against osteosarcoma *in vitro* and *in vivo*.

For a protein to serve as a target for immunotherapy it must elicit a measurable, vigorous and durable immune response. We found NSCLC patient sera contained CTA-specific antibodies, suggesting SP17, AKAP4, and PTTG1 were markedly immunogenic in patients with this disease. Conversely, none of the healthy individuals, or patients whose tumors didn't express CTAs, were found to have circulating anti-CTA antibodies. Shan *et al*. [23] have also reported anti-NY-ESO-1, XAGE-1, ADAM29 and MAGEC1 Abs in the serum of NSCLC patients, but not in healthy individuals. Thus, our findings suggest the potential utility of measuring anti-SP17/AKAP4/PTTG1 antibodies in patients' sera as a marker of immunogenicity against these antigens and, possibly, as a diagnostic tool in NSCLC patients. Since our results are based on a limited series of patients' samples, we believe further analysis is warranted to determine the prevalence and significance of the presence of circulating anti-CTA antibodies in this patient population. Spontaneous CTA-specific immune responses against tumor cells are a feature commonly seen in other malignancies such as recently found in estrogen-receptor negative breast cancers [24], and they might also have an outcome on patients' overall survival, as described by Freitas M, et al., who reported the expression of the CTAs, ACTL8, OIP5, XAGE3 and CTCFL, as an independent predictor of better OS in glioblastoma [25].

To determine if SP17/AKAP4/PTTG1 could trigger a tumor-specific cytotoxic response, we used CTA-loaded DCs to generate CTLs [3, 5, 26, 27]. For each antigen, we selected patients that tested positive for SP17, AKAP4, and PTTG1 protein expression in tumor cells. As expected, patients' PBMCs stimulated with CTA-loaded DCs displayed significant lytic activity against autologous NSCLC cells. The observed cytotoxic effect was clearly HLA class I-restricted and CTA-specific. Cytokine expression analysis revealed that our DC-based CTL generation protocol induced a strong effector polarization in stimulated PBMCs, as evidenced by increased IFN-γ and TNF-α levels, but no changes in tolerogenic IL-4, IL-5, and IL-10 production [3, 11, 27-29]. Since NSCLC patients have been shown to have an abnormal Th-2-type cytokine pattern [30] which negatively impacts immunotherapy [31], we believe the ability of CTA-loaded DCs to stimulate Th-1 polarization effect may be clinically relevant [32]. The activation of effector CTLs was further demonstrated by IFN-γ ELISPOT analysis. This finding is relevant since increased IFN-γ by CTLs has been shown to exert significant inhibitory effects on NSCLC growth [33].

## CONCLUSIONS

The feasibility of generating an active, CTA-specific immune response *in vitro* indicates the expression of the CTAs Sp17, AKAP-4 and PTTG1 by NSCLC cells could have therapeutic relevance and serve as the basis for designing novel immunotherapeutic strategies against this disease. We believe that DCs engineered *in vitro* to recognize and present specific CTAs, such as SP17, AKAP4 and PTTG1, may result in activation of Th1-polarized CTLs capable of overcoming the immunosuppression [31] seen in the NSCLC microenvironment. Therefore, a therapeutic strategy that targets these specific CTAs may result in the development of effective DC-based vaccines against NSCLC [34]. Further studies are necessary to determine the optimal way to generate CTA-specific CTL responses *in vivo*, in order to overcome the immune tolerance observed in this disease [35].

## MATERIALS AND METHODS

### Cells lines

The NSCLC cell lines CRL-5928 (squamous cell carcinoma), CRL-5922 (adenocarcinoma), and the immortalized, non-tumorigenic human bronchial epithelial cell line CRL-2503 (established by transfection with the origin of replication-defective SV40 large T plasmid), were obtained from the American Type Culture Collection (ATCC, Manassas, VA, USA) and maintained according to ATCC guidelines at 37 °C and 5% CO_2_.

### Patients and Clinical Materials

We evaluated a total of 57 NSCLC samples and 16 non-tumor samples, as follows. For quantitative RT-PCR (qRT-PCR), we analyzed 40 NSCLC- and 8 normal lung– derived cDNA samples obtained from OriGene (Table 1, cat. # HLRT301, OriGene, Rockville, MD, USA). For flow-cytometry, ELISA, and cytotoxicity assay, we used 17 NSCLC biopsy samples (median patient's age = 66 years) derived from patients (Table 2) and 8 samples from healthy subjects (no history of smoking, median age = 59 years, 5 females, 3 males) who underwent bronchial biopsy. Sera were collected at the time of routine blood tests as previously described[3]. Materials from human subjects were obtained with approval of the Texas Tech University HSC IRB (L-Micro Study, IRB NUMBER: L04-095), and the patients' written informed consent. Biopsies were minced and plated in RPMI-1640 supplemented with 10% FBS, 20 mM HEPES buffer, 100 U/mL penicillin, 100 mg/mL streptomycin. Cells were maintained in 5% CO_2_ and 37 °C for 16 hours prior to analysis.

**Table 1:** Characteristics of the samples evaluated by qRT-PCR + or − indicates the PCR was positive or negative for the indicated CTA (SP17, AKAP4, or PTTG1). +/−= weak expression.

Histology (stage)	sex	age	Stage	SP17	AKAP4	PTTG1
**Within normal limits**	Male	64	n/a	−	−	−
**Within normal limits**	Female	49	n/a	−	+/−	−
**Within normal limits**	Male	79	n/a	−	−	−
**Within normal limits**	Female	70	n/a	−	−	−
**Within normal limits**	Female	62	n/a	−	−	−
**Within normal limits**	Male	49	n/a	−	−	−
**Within normal limits**	Male	70	n/a	−	−	−
**Within normal limits**	Male	72	n/a	+	−	−
**Carcinoma of lung, squamous cell**	Female	75	IA	−	−	−
**Tumor of lung, carcinoid**	Female	72	IA	+	−	−
**Carcinoma of lung, squamous cell**	Male	71	IA	−	−	+
**Carcinoma of lung, adenosquamous**	Male	66	IA	−	−	−
**Carcinoma of lung, squamous cell**	Male	79	IB	+	−	−
**Carcinoma of lung, large cell**	Male	79	IB	+	−	+
**Carcinoma of lung, adenosquamous**	Female	68	IB	+	−	−
**Adenocarcinoma of lung**	Male	64	IB	−	+	−
**Carcinoma of lung, non-small cell**	Male	65	IB	+	+	−
**Carcinoma of lung, non-small cell**	Female	70	IB	−	−	−
**Carcinoma of lung, large cell**	Male	81	IB	+	−	−
**Carcinoma of lung, squamous cell**	Female	85	IB	−	−	−
**Carcinoma of lung, squamous cell**	Male	76	IB	−	−	−
**Carcinoma of lung, sarcomatoid**	Female	66	IB	+	+	−
**Carcinoma of lung, non-small cell**	Male	71	IB	−	−	−
**Carcinoma of lung, squamous cell**	Male	66	IB	+	−	−
**Adenocarcinoma of lung**	Female	62	IB	+	−	−
**Carcinoma of lung, squamous cell**	Male	72	IB	−	−	−
**Adenocarcinoma of lung**	Female	64	IB	−	−	−
**Carcinoma of lung, large cell**	Male	66	IB	+	+	−
**Carcinoma of lung, large cell, neuroendocrine**	Male	58	IIA	−	−	−
**Carcinoma of lung, squamous cell**	Female	68	IIA	−	+	+
**Carcinoma of lung, non-small cell**	Male	63	IIB	−	−	−
**Carcinoma of lung, squamous cell**	Male	77	IIB	+	−	−
**Adenocarcinoma of lung**	Female	55	IIB	+	+	+
**Adenocarcinoma of lung, acinar, papillary**	Female	77	IIB	−	−	−
**Carcinoma of lung, large cell, neuroendocrine**	Male	62	IIB	−	+	−
**Adenocarcinoma of lung**	Female	71	IIB	−	−	+
**Carcinoma of lung, squamous cell**	Male	71	IIB	−	+	−
**Carcinoma of lung, squamous cell**	Male	76	IIB	−	+	+
**Adenocarcinoma of lung**	Male	81	IIB	−	+	−
**Carcinoma of lung, squamous cell**	Female	66	IIB	+	−	−
**Carcinoma of lung, large cell**	Male	72	IIB	−	−	+
**Adenocarcinoma of lung, bronchioloalveolar**	Female	38	IIB	−	−	−
**Carcinoma of lung, squamous cell**	Male	58	IIIA	+	−	+
**Carcinoma of lung, squamous cell**	Male	62	IIIA	−	+	−
**Adenocarcinoma of lung, papillary**	Male	66	IIIA	+	−	−
**Adenocarcinoma of lung**	Male	80	IIIB	−	−	−
**Carcinoma of lung, squamous cell**	Male	57	IIIB	−	−	−
**Carcinoma of lung, non-small cell, metastatic**	Male	51	IV	−	+	−

**Table 2:** Characteristics of the NSCLC patient's cohort evaluated by FC, IF, and ELISA + or − indicates the tumor cells were positive or negative for the indicated CTA (SP17, AKAP4, or PTTG1), or for the CTA-specific IgG (n.a.= serum not available).

Histology (stage)	sex	age	smoke history?	SP17	AKAP4	PTTG1	SP17-IgG	AKAP4-IgG	PTTG1-IgG
**Squamous cell carcinoma (IIIA)**	F	79	yes	−	−	+	−	−	+
**Adenocarcinoma**	M	74	no	−	−	+	−	−	+
**Squamous cell carcinoma (IV)**	M	76	no	+	−	−	+	−	−
**Large cell carcinoma NOS**	M	49	yes	+	−	+	+	−	+
**Adenocarcinoma (II)**	M	58	yes	+	−	−	+	−	−
**Non-small cell carcinoma (I)**	M	67	yes	+	−	−	+	−	−
**Adenocarcinoma (IIIA)**	M	65	yes	−	+	+	−	+	+
**Carcinoma (IIIA)**	F	75	yes	+	+	−	n.a.	n.a.	n.a.
**Squamous cell carcinoma (II)**	F	71	yes	+	+	−	+	+	−
**Adenocarcinoma (IIIB)**	F	59	yes	+	−	+	+	−	+
**Adenocarcinoma (IV)**	M	66	no	−	−	+	−	−	+
**Non-small cell carcinoma (IIIB)**	F	80	yes	−	−	−	−	−	−
**Squamous cell carcinoma (IIIA)**	M	58	yes	−	+	−	−	+	−
**Adenocarcinoma (II)**	M	51	yes	+	−	−	n.a.	n.a.	n.a.
**Adenocarcinoma (IIIB)**	M	50	yes	−	+	−	−	+	−
**Bronchiolo-alveolar carcinoma non-mucinous (II)**	F	70	yes	+	−	−	n.a.	n.a.	n.a.
**Adenocarcinoma (IIIA)**	F	64	yes	−	−	−	−	−	−

### qRT-PCR

qRT-PCR analysis was performed as previously described[3], using an iCycler iQ Real Time PCR machine SYBR ® Green I Supermix (both from Bio-Rad Laboratories, Inc). The primers sequences were: SP17: 5′-GCTCGGAGAGAAAGGAGGTTC-3′; 5′-TACTCCCCCATTCTGCTGGA-3′; AKAP4: 5′-CAGTCAAGGCTGTAGGAGGG-3′ and 5′-AGCATATCACTTTCCGGTCC-3′; PTTG1: 5′-AGTACTTGTTGGCTCACGCC-3′ and 5′-AGGAGACTGCAACAGATTGGA-3′; β-actin: 5′-CGT CTT CCC CTC CAT CG-3′ and 5′-CTC GTT AAT GTC ACG CAC-3′. Values were expressed by the 2^−δCt^ formula, where ΔCt= Ct housekeeping (β-actin)-Ct gene.

### Immunocytochemistry (ICC)

Following tryspinization for 5 minutes, cell lines were suspended in PBS and spun down on a microscope slide (10,000/spot), then the fixed with 2% formaldehyde in PBS. The staining procedure was performed at room temperature using the same protocol for tissues and cell lines, as follows. Endogenous peroxidase blockade was performed in 0.3% V/V H_2_O_2_ for 5 minutes. Following permeabilization with 0.15% V/V Triton-X100 in PBS, samples were rinsed in PBS and blocked with blocking buffer (5% FBS + 0.05% Tween-20 in PBS) for 1 hour, then incubated with the primary antibody (10 μg/mL in blocking buffer) for 1 hour. After 3 washing steps in blocking buffer (5 minutes each), samples were incubated with the corresponding secondary antibody (2 μg/mL in blocking buffer) for 1 hour. HRP-DAB reaction was carried out for 5 minutes after 3 additional washes, and stopped in water. Slides were mounted with a glycerol-based medium before taking pictures with an inverted Olympus X71 microscope. Antibodies were as follows: rabbit anti-SP17 and mouse anti-PTTG1 (Abcam), goat anti-AKAP4 (Santa Cruz). All secondary antibodies (HRP-linked) were from Abcam.

### Flow-cytometry

400,000 cells were washed with PBS 1X and fixed with buffered paraformaldehyde (4% W/V in PBS, pH=7.4). After 5-min permeabilization on ice with permeabilization buffer (0.3 % V/V saponin in PBS), cells were incubated with specific primary Abs: rabbit anti-human SP17, goat anti-human AKAP4 (Santa Cruz Biotechnology, Santa Cruz, CA, USA), or rabbit anti-human PTTG1 (Novus Biologicals, CO, USA). Negative controls were cells incubated with equal amounts of isotype-matching unspecific IgG immunoglobulins (Novus Biologicals, LLC, Littleton, CO, USA). After 1-h incubation on ice, cells were washed three times in permeabilization buffer, and then incubated on ice (20 minutes) with the appropriate FITC-conjugated secondary antibody (BD Biosciences, CA, USA). Following 3 washing steps with permeabilization buffer, cells were resuspended in PBS and analyzed using a FACS-Canto flow cytometer (BD).

### Immunofluorescence

Immunofluorescence was performed as previously described[12]. Briefly, 20,000 cells were subjected to cytospin and fixed with SlideRite (Fisher). Each sample was permeabilized in 0.1% Triton X-100 sodium citrate buffer for 15 minutes at 4 °C. Cells were then incubated overnight at 4 °C with the specific primary antibodies as described in the flow-cytometry method (1:100 dilution) and then with FITC-conjugated rabbit IgG secondary antibodies (1:500 dilution, Abcam). Standard DAPI staining was used to detect the nuclei. Staining was observed with an inverted fluorescence microscope (Olympus IX71), and representative images were captured at 60X and 10X magnification.

### ELISA for detection of CTA-specific antibodies

Polystyrene 96-well plates were coated with recombinant proteins (SP17, AKAP4 or PTTG1, from Novus Biologicals, LLC, Littleton, CO, USA) in 50 μL carbonate coating buffer (10 μg/well) for 2 hours at 25 °C. After washing twice with 100 μL PBS, plates were blocked with 1% W/V BSA in PBS for 1 hour. BSA was then removed and 50 μL sera (diluted 1:5 in PBS) were added to plates. After 1 hour incubation at 34 °C, plates were washed twice with 100 μL washing buffer (0.05% V/V Tween-20 in PBS), and incubated with HRP-linked mouse anti-human IgG (Abcam, diluted 1:4,000 in PBS). After 1-hr incubation in the dark plates were washed twice with 100 μL washing buffer and then incubated with HRP substrate solution (TMB) in the dark for 5 minutes at 22 °C. Absorbance was read at 450 nm after incubation with 1N HCl. Absorbance of negative controls (antigen-free wells incubated with commercial antibodies) was subtracted to that obtained in wells incubated with sera.

### Isolation of PBMCs and Generation of Dendritic Cells (DCs)

Heparinized blood was centrifuged in a Ficoll-Hypaque gradient to separate PBMCs, which were then incubated in RPMI-1640 medium (3×10^6^ cells/mL) for 2 hours. Non-adherent cells were removed and adherent cells were cultured in complete RPMI-1640 supplemented with 1000 IU/mL IL-4 and 800 IU/mL GM-CSF. After 1 week, DCs were harvested and pulsed with human recombinant SP17, AKAP4 or PTTG1 as described [3].

### DC Pulsing

DCs were washed twice and placed in a 50 mL polypropylene tube. Recombinant proteins were mixed with the cationic lipid DOTAP (Roche, Mannheim, Germany) for 20 minutes at RT, and added to the DCs for 3 hours at 37 °C.

### Generation of CTA-specific Cytotoxic T Lymphocytes (CTLs), *in vitro*

Antigen-pulsed DCs were co-cultured with fresh autologous PBMCs [3] at a ratio of 1:10 in RPMI-1640 supplemented with 10% autologous serum, 10 IU/mL IL-2 and 5 ng/mL IL-7 at 37°C, 5% CO_2_. Irradiated autologous PBMCs feeder cells and recombinant CTAs (50 μg/mL) were added once a week, and IL-2 was added every 3 days. Recombinant CTAs were endotoxin-free, as confirmed by endotoxin detection assay performed through the ToxinSensor Chromogenic LAL Endotoxin Assay Kit (GenScript USA Inc., NJ).

### Cytotoxicity essay

We performed an EUROPIUM-based cytotoxicity assay using the DELFIA^®^ EuTDA system according to the manufacturer's instructions. Target cells were autologous tumor cells (at the effector-target cell ratios of 40:1, 20:1, or 5:1). Antibodies against HLA class I (W6/32) and HLA class II (L243) were added (25 μg/mL) to evaluate HLA restricted cytotoxicity, with a fixed 20:1 (effector:target) ratio. Experiments were run in triplicates.

### ELISA

ELISA was performed on the supernatants of activated PBMCs and autologous tumor cells 4-hour co-cultures (20:1 effector:target ratio). The U-CyTech sandwich ELISA kit (U-CyTech, Utrecht, The Netherlands) was used in accordance with the manufacturer's instructions. Reactions were developed by adding the TMB substrate and stopped by the addition of 2 M H_2_SO_4_. The absorbance was read at 450 nm. Data are presented as the optical densities (ODs) measured with activated PBMCs divided by the ODs obtained with PBMCs incubated with autologous DCs without antigens (OD ratio).

### ELISPOT

IFN-γ expression was evaluated using ELISPOT assay (UCyTech, Utrecht, The Netherlands), as previously described [3]. Spots were counted with an AID ELISPOT Reader System (Cell Technology, Inc., Columbia, MD).
